# CGSEA: A Flexible Tool for Evaluating the Associations of Chemicals with Complex Diseases

**DOI:** 10.1534/g3.119.400945

**Published:** 2020-01-14

**Authors:** Shiqiang Cheng, Mei Ma, Lu Zhang, Li Liu, Bolun Cheng, Xin Qi, Chujun Liang, Ping Li, Om Prakash Kafle, Yan Wen, Feng Zhang

**Affiliations:** Key Laboratory of Trace Elements and Endemic Diseases of Ministry of Health, School of Public Health, Health Science Center, Xi’an Jiaotong University, Xi’an, P. R. China

**Keywords:** complex diseases, chemicals, genome-wide association study, gene set enrichment analysis

## Abstract

The etiology of many human complex diseases or traits involves interactions between chemicals and genes that regulate important physiological processes. It has been well documented that chemicals can contribute to disease development through affecting gene expression *in vivo*. In this study, we developed a flexible tool CGSEA for scanning the candidate chemicals associated with complex diseases or traits. CGSEA only need genome-wide summary level data, such as transcriptome-wide association studies (TWAS) and mRNA expression profiles. CGSEA was applied to the GWAS summaries of attention deficiency/hyperactive disorder, (ADHD), autism spectrum disorder (ASD) and cervical cancer. CGSEA identified several significant chemicals, which have been demonstrated to be involved in the development or treatment of ADHD, ASD and cervical cancer. The CGSEA program and user manual are available at https://github.com/ChengSQXJTU/CGSEA.

The pathogenesis of many human complex diseases or traits arise from interactions between environmental factors and genes that regulate important physiological processes (Olden and Wilson 2000). Chemicals in the environment play critical roles in the etiology of many human complex diseases. For example, benzene is a ubiquitous chemical in our living environment. It can cause acute leukemia and other hematological cancers (Smith 2010). Arsenic contributes to the development of diabetes (Navasacien *et al.* 2005). However, the traditional methods used to explore the interactions between chemicals and complex diseases have some limitations, such as elucidating the molecular mechanisms of action of environmental chemicals, developing methods to predict toxicity effectively and understanding the genetic basis of differential susceptibility (Mattingly *et al.* 2004). In addition, environmental exposure of chemicals is usually mixed. Therefore, it is difficult to accurately measure exposure levels *in vivo*.

It has been well documented that chemicals generate biological effects through affecting gene expression *in vivo*. For example, previous study have observed gene expression changes that were associated with occupational benzene exposure in the peripheral blood mononuclear cell, such as *CXCL16*, *ZNF331*, *JUN* and *PF4* (McHale *et al.* 2009). A previous study suggested that the expression of dispersed genes may be prone to environmental stimuli while that of clustered genes may be resistant and concluded that environmental components were able to account for most of the positional variation in gene expression changes (Choi and Kim 2007). Comparative Toxicogenomics Database (CTD) is a well-known database, which includes extensive annotations of associations between chemicals and gene expression. CTD was established based on the published high-throughput experimental data and curate toxicologically important genes. By searching references with multiple, large vocabularies, the contributors are compiling a more comprehensive literature set that is relevant to the effects of chemicals on gene expression (Mattingly *et al.* 2006).

Genome-wide association study (GWAS) methodology has advanced such that it is now a powerful tool for the dissection of more complex genetic architectures of human diseases or traits (Mccarthy *et al.* 2008). It is well known that gene expression is under genetic control and a large part of the candidate loci identified by GWAS affect diseases by regulating gene expression (Dimas *et al.* 2009). This motivates the development of transcriptome-wide association studies (TWAS), which is a promising approach to evaluate the expression associations of gene with complex diseases or traits by only using GWAS summaries and take tissue specificities into consideration (Gusev *et al.* 2016). For instance, Gusev *et al.* used TWAS and identified 69 new genes associated with obesity-related traits in blood and adipose tissue (Gusev *et al.* 2016). In this study, based on the chemical-gene interaction networks, we developed a flexible tool CGSEA, which is capable to detect the associations between chemicals and complex diseases or traits utilizing high-throughput omics summary statistics (such as TWAS and mRNA expression profiles). We applied CGSEA to publicly available GWAS summaries of attention deficiency/hyperactive disorder (ADHD), autism spectrum disorder (ASD) and cervical cancer to illustrate the good performance of CGSEA.

## Methods

### Implementation

#### Step1 - Chemical-gene expression annotation dataset:

The relationships between chemicals and gene expression changes were obtained from the CTD (http://ctdbase.org/downloads/), including organic chemicals, polycyclic compounds, biological factors and enzymes and coenzymes. Currently, CTD provides over 1,929,106 interactions between 13,151 chemicals and 48,092 genes in 591 organisms. Specific for this study, a total of 1,788,149 annotation terms of chemical-gene pairs driven from human and mice were used in this study. We finally generated 11,190 chemicals related gene sets. The overview of the information retrieval process of CTD can be found in the previous study (Mattingly *et al.* 2006).

#### Step2 –Gene expression association testing statistics of complex diseases:

In this study, we used the TWAS expression association testing statistics (TWAS *Z-score*) calculated by the FUSION software (http://gusevlab.org/projects/fusion/) (Gusev *et al.* 2016). First, TWAS was conducted to test the associations between target diseases and the gene expression levels imputed by the prediction models of FUSION (Gusev *et al.* 2016). Briefly, for a given gene, the SNP-expression weights in the 1-Mb cis loci of the gene were first computed with the Bayesian sparse linear mixed model (BSLMM) (Zhou *et al.* 2013). The imputed gene expression data can be viewed as a linear model of genotypes with weights based on the correlation between SNPs and gene expression in the training data while accounting for linkage disequilibrium (LD) among SNPs (Gusev *et al.* 2016). The gene expression weights were then combined with summary-level GWAS results to calculate the association statistics between gene expression levels and each of the disease. Specific for this study, the expression weights of brain RNA-seq and whole blood RNA array were downloaded (http://gusevlab.org/projects/fusion/), and used as reference data in the TWAS of ASD and ADHD. The expression weights of cervical squamous cell carcinoma RNA-seq from The Cancer Genome Atlas (TCGA) and whole blood RNA array were downloaded (http://gusevlab.org/projects/fusion/), and used as reference data in the TWAS of cervical cancer. The expression weights reference data of brain RNA-seq, whole blood RNA array and cervical squamous cell carcinoma RNA-seq contain 5419, 4700 and 1117 genes respectively. Let $L_{\text{i}}^{}$denote the TWAS statistic (Z-score) of the *i*th gene. All genes are ranked by sorting $L_{z\text{i}}^{}$from maximum to minimum ($L_{1}^{s}\geq L_{2}^{s}\geq ....L_{\text{n}}^{s}$), denoted as$L_{}^{s}=[L_{1,}^{s}L_{2}^{s},....L_{\text{n}}^{s}]$. Additionally, the gene expression association testing statistics can also be driven from gene expression profile studies.

#### Step3 – Chemical related gene set enrichment analysis:

For a given chemical related gene set C with $N_{C}^{}$ genes, let $g_{i}^{}$denotes the i*th* gene of the gene set C. Let $ES_{}^{C}$ denote the enrichment scores (ES) of gene set C, which was calculated by weighted Kolmogorov–Smirnov-like running sum statistic in gene set enrichment analysis (GSEA) (Subramanian *et al.* 2005, Wang *et al.* 2007), defined by

$$ES_{}^{C}=\underset{1\leq j\leq N}{\max}\left\{\sum_{g_{i}\in C,i\leq j}\frac{|L_{{}_{i}}^{\text{s}}{|}^{w}}{N_{R}}-\sum_{g_{i}\notin C,i\leq j}\frac{1}{N-N_{C}}\right\},$$

where $N_{R}=\sum_{g_{i}\in C}|L_{{}_{i}}^{s}{|}^{w}$. *N* denotes the total number of genes. *w* is a parameter giving higher weights to genes with extreme statistics. *j* denotes the gene set size (number of genes) related to a given chemical. CGSEA calculates the *ES^C^* by walking down the ranked list L of genes, increasing a running-sum statistic when a gene is in the gene set C and decreasing it when it is not. Without loss of generality, *w* was assigned to be 1 in this study. For statistic tests, permutations were conducted to obtain the null distribution of $ES_{}^{C}$(denoted as $ES_{null}^{\text{C}}$), through randomly shuffling the gene labels. Let $ES_{tnull}^{\text{C}}$ denote the ES value of gene set C of *t*th permutation. After *P* times permutations, we obtained the null distribution of$ES_{null}^{\text{C}}$, denoted as $ES_{null}^{\text{C}}=\left[ES_{1null}^{C},ES_{2null}^{C},\ldots ..ES_{pnull}^{C}\right]$. The observed ES ($ES_{}^{C}$) of the gene set C was normalized by the mean value and standard deviation of permutated ES ($ES_{null}^{\text{C}}$), defined by

$$NES_{}^{C}=\frac{ES_{}^{C}-mean\left(ES_{null}^{C}\right)}{SD\left(ES_{null}^{C}\right)}$$

Where $NES_{}^{C}$denoted the normalized ES of the gene set C. Let $NES_{null}^{C}$ denoted the null distribution of $NES_{}^{C}$. $NES_{null}^{C}$ was defined as $NES_{null}^{C}=\left[NES_{1null}^{C},NES_{2null}^{C},\ldots ..NES_{pnull}^{C}\right]$, which could be calculated from *P* permutations using the similar formula:

$$NES_{inull}^{C}=\frac{ES_{inull}^{C}-mean\left(ES_{null}^{C}\right)}{SD\left(ES_{null}^{C}\right)}$$

**Figure 1:**
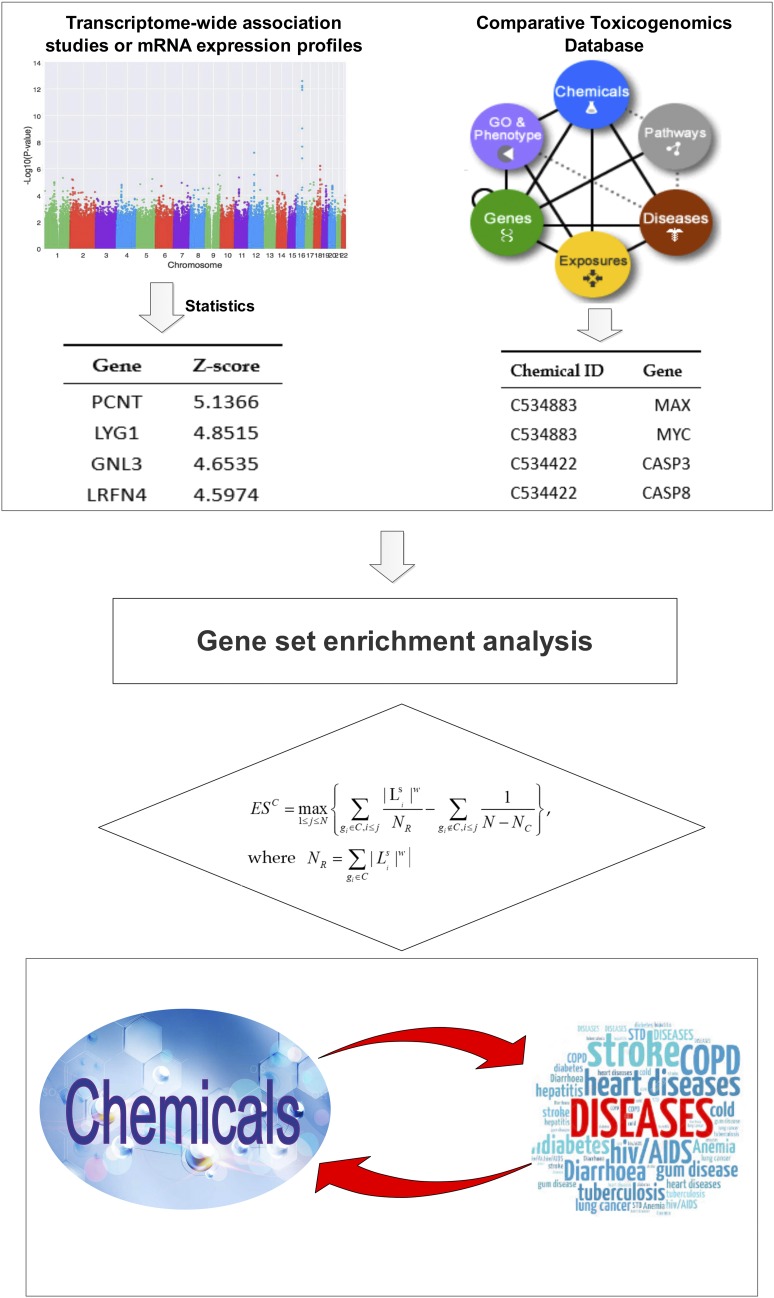
The general analytical procedures of CGSEA.

For the given chemical related gene set C, the empirical *P* were calculated from the observed $NES_{}^{C}$ and $NES_{null}^{C}$ following the widely used approach (Wang *et al.* 2007, Wen *et al.* 2016). We developed a tool CGSEA to implement the approach proposed by this study. In GSEA, the gene sets are defined based on prior biological knowledge, such as published information about biochemical pathways or coexpression in previous experiments (Subramanian *et al.* 2005). In addition, the traditional GSEA usually obtained the gene expression statistics from mRNA expression profiles. In CGSEA, a gene set is any group of genes that share a particular chemical, and the aim is to determine whether that chemical has a role in the phenotype of interest. Meanwhile, the gene expression statistics were computed by TWAS, which is not susceptible to the environmental confounders that may influence expression. However, despite those differences, the underlying statistical structure (weighted Kolmogorov–Smirnov-like statistic) is essentially the same. To facilitate the application of CGSEA, the CTD chemical-gene annotation file used by this study has been included in the CGSEA package. Figure 1 presents the general analytical procedures of CGSEA.

### Application to ADHD, ASD and cervical cancer

The GWAS summaries of ADHD (19 099 cases and 34 194 controls) and ASD (7 387 cases and 8 567 controls) were downloaded from the Psychiatric GWAS Consortium (PGC) website (https://www.med.unc.edu/pgc/ results-and-downloads). The GWAS summaries of carcinoma *in situ* of cervix uteri were derived from the UK Biobank, including 1 992 patients and 243 502 healthy controls of European ancestry. TWAS was conducted by the FUSION software (http://gusevlab.org/projects/fusion/) (Gusev *et al.* 2016). For the TWAS of ADHD and ASD, the gene expression weight references of whole blood and brain tissues were used. For the TWAS of cervical cancer, the gene expression reference weight of cervical squamous cell carcinoma was used. 5,000 permutations were conducted by CGSEA in this study.

### Data availability

All data used in this manuscript are freely available and published online. The GWAS summaries of ADHD and ASD can be found on the Psychiatric GWAS Consortium (PGC) website (https://www.med.unc.edu/pgc/ results-and-downloads). The GWAS summaries of carcinoma *in situ* of cervix uteri can be found on the UK Biobank under the accession D06 (http://geneatlas.roslin.ed.ac.uk/downloads/). Supplemental material available at figshare: https://doi.org/10.25387/g3.11604990.

## Results and discussion

Table 1 summarizes the top three significant chemicals identified by CGSEA for ADHD, ASD and cervical cancer, respectively. The functional relevance of some identified chemicals with ADHD, ASD and cervical cancer have been reported by previous study. For example, prenatal exposure to methylazoxymethanol acetate (*P* = 0.0006) lead to alterations in the medial prefrontal cortex indicative of a compromise in information processing (Goto and Grace 2006). Ro-31-8220 (*P* = 0.0010) is one of AKT inhibitors. Chen *et al.* have suggested that IGF-I/PI3K/AKT/mTOR pathway has potency in the diagnosis and treatment of ASD (Chen *et al.* 2014). Antioxidant vitamin (vitamins A, C, and E (*P* = 0.0130)) intake was suggested to decrease the risk of cervical cancer (Kim *et al.* 2010). The increase in the incidence of precancerous lesions of the cervix in areas near the borders with the former Yugoslavia during 1997-1999 may be influenced by environmental factors such as exposure to depleted uranium (*P* = 0.0042) (Papathanasiou *et al.* 2005).

**Table 1:** List of top three chemicals identified by CGSEA for ADHD, ASD and cervical cancer

Disorders	Chemicals	Empirical *P* value
**ADHD**	Crizotinib	0.0002
	Ketoconazole	0.0004
	Methylazoxymethanol Acetate	0.0006
**ASD**	Ptaquiloside	0.0008
	Ro 31-8220	0.0010
	Ethoxyquin	0.0024
**Cervical cancer**	Uranium	0.0042
	4-toluidine	0.0068
	Vitamin E	0.0130

Note: attention deficiency/hyperactive disorder, ADHD; autism spectrum disorder, ASD.

In this study, we developed a flexible tool CGSEA for scanning the candidate chemicals associated with complex diseases or traits. CGSEA has two advantages. First, our approach only need genome-wide summary level data (such as the summaries of TWAS and mRNA expression profiles), which are usually available online for many complex disease and traits. Second, our approach explores the functional association of chemicals and diseases from the genomic perspective, thus the results should be more robust to overcome the shortcomings of traditional methods, such as it is difficult to accurately measure *in vivo* exposure.

The tool of CGSEA is mainly developed for scanning candidate chemicals associated with human complex diseases or traits. Due to the following two reasons, we only used the chemical related gene sets collecting from human and rice. First, many of the organisms included in CTD are invertebrates and non-mammal, such as cnidarians and ctenophores. Due to different genetic background, it is difficult to generalize the results to other organisms. Second, the mouse has a long and rich history in biological research, and many consider it a model organism for the study of human development and complex disease (Pennisi 2002, Bogue 2003). Therefore, we used the chemical related gene sets collecting from human and mice in this study.

However, two limitations of this approach should be noted. First, the performance of CGSEA may be affected by the accuracy of TWAS results and chemical related gene sets. Second, all subjects in this study are from European ancestry. Due to different genetic background, our study results should be interpreted with caution when applied to other populations.
